# Geographic distribution and molecular analysis of porcine reproductive and respiratory syndrome viruses circulating in swine farms in the Republic of Korea between 2013 and 2016

**DOI:** 10.1186/s12917-018-1480-6

**Published:** 2018-05-16

**Authors:** Hyeonjeong Kang, Ji Eun Yu, Ji-Eun Shin, Areum Kang, Won-Il Kim, Changhee Lee, Jienny Lee, In-Soo Cho, Se-Eun Choe, Sang-Ho Cha

**Affiliations:** 1 Viral Disease Division, Animal and Plant Quarantine Agency, Gimcheon, 39660 Republic of Korea; 2 Animal Virology Laboratory, School of Life Sciences, Kyungpook National University, Daegu, 41566 Republic of Korea; 3 College of Veterinary Medicine, Jeonbuk National University, Iksan, 54596 Republic of Korea; 4 Present address: PRRS research Laboratory, Viral Diseases Division, Animal and Plant Quarantine Agency, Gimcheon, 39660 Republic of Korea

**Keywords:** Porcine reproductive and respiratory syndrome virus (PRRSV), Genetic diversity, Open reading frame 5 (ORF5) sequence, Phylogenetic analysis

## Abstract

**Background:**

Porcine reproductive and respiratory syndrome virus (PRRSV) causes devastating disease characterized by reproductive failure and respiratory problems in the swine industry. To understand the recent prevalence and genetic diversity of field PRRSVs in the Republic of Korea, open reading frames (ORFs) 5 and 7 of PRRSV field isolates from 631 PRRS-affected swine farms nationwide in 2013–2016 were analyzed along with 200 Korean field viruses isolated in 2003–2010, and 113 foreign field and vaccine strains.

**Results:**

Korean swine farms were widely infected with PRRSVs of a single type (38.4 and 37.4% for Type 1 and Type 2 PRRSV, respectively) or both types (24.2%) with up to approximately 83% nucleotide sequence similarity to prototype PRRSVs (Lelystad or VR2332). Phylogenetic analysis based on the ORF5 nucleotide sequence revealed that Korean Type 1 field isolates were classified as subgroups A, B, and C under subtype 1, while Korean Type 2 field isolates were classified as lineages 1 and 5 as well as three Korean lineages (kor A, B, and C) with the highest infection prevalence in subgroup A (50.5%) and lineage 5 (15.3%) for Type 1 and Type 2 PRRSV, respectively, among ORF5-positive farms. In particular, the lineages kor B and C were identified as novel lineages in this study, and lineage kor B comprised only the field viruses isolated from Gyeongnam Province in 2014–2015, establishing regionally unique genetic characteristics. It has also recently been confirmed that commercialized vaccine-like viruses (subgroup C) of Type 1 PRRSV and NADC30-like viruses of Type 2 PRRSV (lineage 1) are spreading rapidly in Korean swine farms. The Korean field viruses were also expected to be antigenically variable as shown in the high diversity of neutralizing epitopes and N-glycosylation sites.

**Conclusions:**

This up-to-date information regarding recent field PRRSVs should be taken into consideration when creating strategies for the application of PRRS control measures, including vaccination in the field.

## Background

Porcine reproductive and respiratory syndrome virus (PRRSV) is one of the most common and economically significant infectious diseases in the pig industry worldwide [1, 2]. This virus causes clinical disease characterized by reproductive failure in pregnant sows and acute respiratory illness in growing pigs, causing increased pre-weaning mortality [3, 4]. PRRSV is a small-enveloped, single-stranded, non-segmented, and positive-sense RNA virus that is a member of the genus *Arterivirus* in the family Arteriviridae and order Nidovirales [5–7] he PRRSV genome is approximately 15 kb in size, composed of a 5′-untranslated region (UTR), 10 open reading frames (ORFs), designated ORF1a, ORF1b, ORF2a, ORF2b, and ORF3–7 including ORF5a, and a 3′-UTR [8–11]. ORF1a and ORF1b encode replication-related polymerase proteins that can be autoproteolytically cleaved into at least 16 nonstructural proteins (NSPs) [12–16]. ORFs 2a through 7 encode the following viral structural proteins: six envelope-associated proteins (GP2a, E, GP3, GP4, GP5, and M) and the nucleocapsid (N) protein [11, 17–19]. ORF5 encodes the major viral envelope protein GP5. As the most variable structural gene of PRRSV, GP5 plays an important role in viral assembly, infectivity, and the induction of neutralizing antibodies [20–23]. Owing to its high degree of genetic diversity, the ORF5 sequence has been used for diagnostic identification and classification of PRRS field viruses [4, 24–26].

PRRSV is divided into two major genotypes: Type 1 (European type) and Type 2 (North American type) PRRSV. Lelystad and VR2332 are considered reference strains of Type 1 and Type 2 PRRSV, respectively [27]. Type 1 and Type 2 PRRSV exhibit approximately 60% genomic sequence identity, and 20% nucleotide sequence variability within each genotype [28, 29]. Owing to the emergence of highly diverse field viruses, global phylogeny using the ORF5 sequence is characterized by many groups (subtypes 1–4 for Type 1 PRRSV and lineages 1–9 for Type 2 PRRSV) within each of the genotypes, composed of genetically and geographically distinct PRRSVs [30, 31].

Previous studies have also reported the genetic diversity and phylogeny of PRRSVs circulating in the Republic of Korea [32–34]. Type 2 PRRSV has infected swine farms since the mid-1980s [35], while Type 1 PRRSV has spread rapidly since its first detection in 2005 [33]. However, there has been no study regarding the current prevalence and genetic characteristics of PRRSV in PRRS-affected swine farms nationwide. Recent surveys among swine farmers in the Republic of Korea revealed that over 50% of swine farms have suffered from PRRS, which has been recognized as the most economically important disease in this industry. Combined with the introduction of new modified live vaccines into swine farms over the past three years, the active circulation of field viruses has increased concern regarding the establishment of novel genetic components, and the emergence of antigenic or virulent variants. The following study was conducted to investigate the prevalence, genetic characteristics, and phylogeny of Korean field PRRSVs in recent years (2013–2016).

## Methods

### Study design

Clinical samples (lung tissues and sera) submitted to diagnostic labs (Animal and Plant Quarantine Agency and Jeonbuk National University) were collected from pigs (mostly older than 4 weeks old) with poor growth and respiratory illness between 2013 and 2016. All samples were tested by multiplex reverse transcription-polymerase chain reaction (RT-PCR) for amplification of ORF7 with primers designed to detect Type 1 and Type 2 PRRSV simultaneously. Virus isolation using ORF7-positive samples was attempted in the MARC-145 cell line and porcine alveolar macrophages (PAMs). Selected viral isolates were sequenced for ORF5, and further subjected to genetic analysis and compared with Korean field viruses isolated in 2003–2010, commercial vaccine strains (DV of Porcilis® PRRS, VP-046 of UNISTRAIN® PRRS, Ingelvac PRRS MLV of Ingelvac PRRS® MLV, and P129 of Fostera® PRRS) commonly used in the Republic of Korea, PRRSV prototype strains (VR2332 and Lelystad), and field isolates previously reported from other countries.

### Sample collection

A total of 631 clinical samples (lung tissues and sera) were obtained from swine farms located in provinces nationwide: Gyeonggi (*n* = 107), Chungbuk (*n* = 9), Chungnam (*n* = 27), Jeonbuk (*n* = 69), Jeonnam (*n* = 35), Gyeongbuk (*n* = 61), Gyeongnam (*n* = 307), Gangwon (*n* = 1), and Jeju (*n* = 15) in 2013–2016. The lung tissues were homogenized in Dulbecco’s minimum essential medium (20% *w*/*v*) and filtered (0.45-μm filter). The lung homogenates and sera were stored at − 70 °C until use.

### RT-PCR

Total RNA was extracted from the lung homogenates and sera using the RNeasy mini kit (Qiagen, Hilden, Germany), in accordance with the manufacturer’s instructions. To detect and differentiate between Type 1 and Type 2 PRRSV, samples were first subjected to multiplex RT-PCR using genotype-specific ORF7 primers (Table 1) and a OneStep RT-PCR kit (Qiagen), which can simultaneously amplify PCR products of 398 and 433 base pairs (bp) for Type 1 and Type 2 PRRSV, respectively. The ORF7-positive sera were further subjected to RT-PCR for ORF5 amplification. The ORF5 genes of PRRSV were amplified with two pairs of primers [33] for Type 1 and Type 2 PRRSV (Table 1), generating PCR products of 754 and 716 bp, respectively. The amplification reaction consisted of 2.5 μl of 5× RT-PCR buffer (including 2.5 mM MgCl_2_), 0.2 mM dNTPs, 0.2 μM of each primer, 0.5 μl of the enzyme mix, and 5 μl of RNA extract in a final volume of 25 μl. RT-PCR was performed by reverse transcription for 30 min at 50 °C, termination of reverse transcription for 15 min at 95 °C, followed by 35 cycles of 20 s at 94 °C, 20 s at 55 °C, 30 s at 72 °C, and a final extension of 10 min at 72 °C in a C1000 Thermal Cycler (Bio-Rad, Hercules, CA, USA). The RT-PCR amplicons were analyzed using 1% agarose gel electrophoresis.

**Table 1:** Primer sequences for amplification of ORF5 and ORF7

Type	Primer			Product (bp)
		Position	Sequence	
1	Forward	14,653–14,671 (ORF7)	5’-ATGGCCAGCCAGTCAATCA-3’	ORF7 (398)
	Reverse	15,030-15,050 (3’NCR)	5’-TCGCCCTAATTGAATAGGTGA-3’	
	Forward	13,444–13,461 (ORF4)	5’-AATGAGGTGGGCYACAACC-3’	ORF5 (754)
	Reverse	15,030–15,050 (ORF6)	5’-GCGTGACACCTTAAGGGC-3’	
2	Forward	14,933–14,951 (ORF7)	5’-ATGGCCAGCCAGTCAATCA-3’	ORF7 (433)
	Reverse	15,346–15,365 (3’NCR)	5’-TCGCCCTAATTGAATAGGTGA-3’	
	Forward	13,759–13,778 (ORF4)	5’-CCATTCTGGTGGCAATTTGA-3’	ORF5 (716)
	Reverse	14,455–14,474 (ORF6)	5’-GGCATATATCATCACTGGCG-3’	

### Nucleotide (NT) and amino acid (AA) sequences and phylogenetic tree analysis

Amplified ORF5 products (*n* = 542) were purified using the QIAquick Gel Extraction Kit (Qiagen) and sequenced (Macrogen, Seoul, Korea). Multiple sequence alignment was initially carried out and homology of the NT and AA sequences among the PRRSV isolates was determined with CLC Main Workbench ver. 7.0.3. ORF5 sequences were subjected to analysis of neutralizing epitopes (NEs), putative N-glycosylation sites (Asn-Xaa-Ser/Thr), and phylogenetic trees compared with those of Korean field viruses (*n* = 200) isolated between 2003 and 2010, field isolates of foreign countries (*n* = 109), including PRRSV prototype strains (VR2332 and Lelystad), and vaccine strains (*n* = 4) used in the Republic of Korea, available in the GenBank database. The phylogenetic tree was constructed using MEGA6 software with the neighbor-joining method and bootstrap values were calculated on 1000 replicates.

## Results

### Prevalence of PRRSVs in the Republic of Korea from 2013 to 2016

**Fig. 1:**
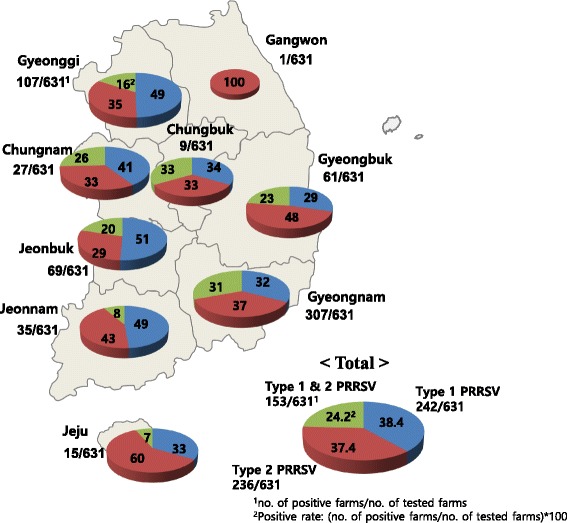
Geographic distribution of Korean field viruses infected in 631 swine farms with respect to single (Type 1 or Type 2 PRRSV) and co-infection (Type 1 & 2 PRRSV)

Among the 631 clinical samples subjected to ORF7 RT-PCR, 38.4% (242/631) were positive for Type 1 PRRSV, 37.4% (236/631) were positive for Type 2 PRRSV, and 24.2% (153/631) were positive for both PRRSV genotypes (Fig. 1). In terms of geographical prevalence by genotype, Type 1 was more prevalent in Gyeonggi and Jeonbuk Provinces, while Type 2 mainly infected in Gyeongbuk Province. Both genotypes were almost equivalently present in the remainder of the provinces.

### Phylogenetic analysis and sequence homology

**Fig. 2:**
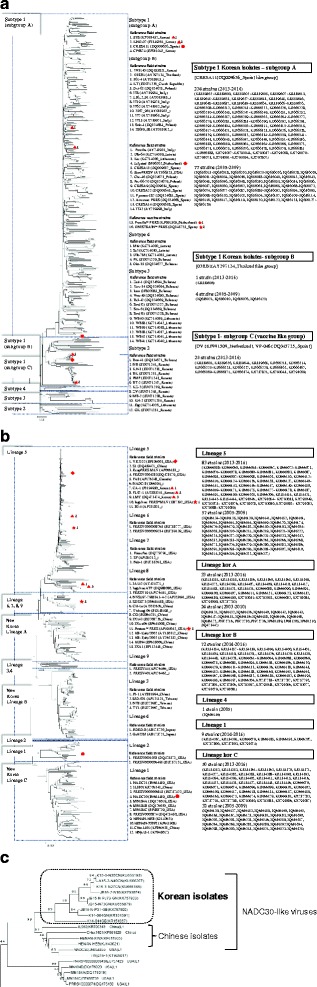
Phylogenetic analysis using ORF5 nucleotide sequences of Type 1 (**a**) and Type 2 PRRS (**b**) field isolates obtained from this study and the GenBank database. The tree was constructed by maximum-likelihood method and the grouping was referred from the previous studies [30, 31, 56]. Prototype viruses of each type, vaccine viruses used in Korean swine farms and Korean reference strains were marked with ◆, ★ and ▲, respectively. CRESA11 was marked with ●. A group of NADC30-like viruses was magnified in (**c**)

Phylogenetic analysis based on global PRRSV phylogeny [30, 31] indicated that all of the Korean isolates (*n* = 542) from 2003 to 2016 analyzed in this study were classified into pan-European subtype 1 for Type 1 PRRSV, and lineages 1, 4, and 5 and three Korean lineages (lineage kor A, B, and C) for Type 2 PRRSV. Regarding the recent Korean Type 1 field viruses (*n* = 295) isolated in 2013–2016, all of the isolates were classified into subgroups A (*n* = 274), B (*n* = 1), and C (*n* = 20) under subtype 1 (Fig. 2a) with infection prevalence rates of 50.5% (274/542), 0.18% (1/542), and 3.69% (20/542) among ORF5-positive farms, respectively. The recent Korean isolates of subgroup A were grouped with 77 Korean field viruses, two Korean reference strains (E38 and KNU-07) isolated in 2005–2009, and a Spanish strain, CRESA11. One isolate of subgroup B was grouped with four Korean field viruses isolated in 2005–2009, a Thai isolate (03RB1), and 12 European reference strains. Subgroup C was formed with only 20 recent Korean isolates, Lelystad, two vaccine strains (DV and VP-046), and 13 European reference strains. Meanwhile, subtypes 2, 3, and 4 were formed with only Eastern European reference strains. NT (AA) sequence similarity to Lelystad was 84.9–89.8% (82.2–91.6%), 85.9% (85.6%), and 93.9–98.4% (90.1–96.5%) in subgroups A, B, and C of field viruses isolated in 2013–2016, respectively. Meanwhile, NT (AA) sequence similarity rates to Lelystad were 87.8–90.9% (86.1–91.6%) and 85.8–86.9% (85.2–89.1%) for subgroups A and B of field viruses isolated in 2005–2009, respectively. In terms of sequence similarity to vaccine strains, NT (AA) sequence similarity to DV was similar to Lelystad for field viruses isolated in 2013–2016 as well as in 2005–2009. NT (AA) sequence similarity to VP-046 was 84.2–88.9% (81.7–92.6%), 85.5% (86.1%), and 93.7–99.8% (90.6–99.5%) in subgroups A, B, and C of field viruses isolated in 2013–2016, while 86.8–89.8% (85.6–91.1%), and 85.5–87.5% (85.2–88.6%) in subgroups A and B of field viruses isolated in 2005–2009, respectively (Table 2A).

**Table 2:** ORF5 nucleotide (NT) and amino acid (AA) sequence similarity of subtype 1/subgroups of type 1 PRRSV (A) and lineages of type 2 PRRSV (B) to prototypes/vaccine strains

Type	Isolated year	Subtype /Lineage	Sequence similarity		
			prototype	Vaccine strain	
			Lelystad virus	DV (Porcilis® PRRS)	VP-046 (UNISTRAIN® PRRS)
(A)					
1	2005-2009	subtype 1-A	87.8-90.9 (86.1-91.6)^a^	87.6-91.1 (86.1-91.6)	86.8-89.8 (85.6-91.1)
		subtype 1-B	85.8-86.9 (85.2-89.1)	85.3-86.8 (84.2-88.1)	85.5-87.5 (85.2-88.6)
	2013-2016	subtype 1-A	84.9-89.8 (82.2-91.6)	85.0-89.6 (82.2-90.6)	84.2-88.9 (81.7-92.6)
		subtype 1-B	86.0 (85.6)	85.5 (84.7)	85.5 (86.1)
		subtype 1-C	93.9-98.4 (90.1-96.5)	93-4-99.5 (89.6-98.5)	93.7-99.8 (90.6-99.5)
Type	Isolated year	Subtype /Lineage	Sequence similarity		
			prototype	Vaccine strain	
			VR2332	Ingelvac PRRS MLV (Ingelvac® PRRS MLV)	P129 (Fostera® PRRS)
(B)					
2	2003-2010	lineage 4	88.9 (88.1) ^b^	88.9 (88.6)	89.7 (89.1)
		lineage 5	88.9-99.5 (88.1-99.0)	89.9-99.8 (87.6-99.5)	88.2-91.9 (88.1-92.5)
		lineage kor A	87.6-89.6 (82.6-88.6)	86.7-89.7 (84.1-91.5)	87.2-90.2 (86.1-93.0)
		lineage kor C	84.7-87.1 (84.1-88.6)	84.7-87.1 (83.6-88.1)	85.4-88.1 (84.1-89.1)
	2013-2016	lineage 1	82.3-86.9 (81.7-87.6)	84.9-86.1 (83.1-85.6)	85.2-86.6 (84.6-86.6)
		lineage 5	87.6-99.3 (85.6-99.0)	87.9-99.7 (87.1-99.5)	86.7-91.7 (84.6-91.5)
		lineage kor A	85.1-89.1 (82.1-87.6)	85.4-88.7 (82.6-88.1)	85.6-89.2 (85.1-90.1)
		lineage kor B	85.7-89.1 (83.6-90.1)	85.7-89.1 (83.1-89.6)	86.1-89.1 (85.1-89.1)
		lineage kor C	85.2-86.4 (83.6-86.1)	83.6-87.6 (81.2-87.1)	82.6-86.9 (81.6-87.6)

^a^NT and AA sequence similarity to Type 1 PRRSV prototypes (Lelystad) and vaccine strains (DV and VP-046). Parenthesis indicates AA sequence similarity

^b^NT and AA sequence similarity to Type 2 PRRSV prototypes (VR2332) and vaccine strains (Ingelvac PRRS MLV, P129). Parenthesis indicates AA sequence similarity

The Korean Type 2 field viruses (*n* = 247) isolated in 2013–2016 were classified into five different lineages: lineage 1 (*n* = 9), lineage 5 (*n* = 83), lineage kor A (*n* = 33), lineage kor B (*n* = 72), and lineage kor C (*n* = 50) (Fig. 2b), with infection prevalence rates of 1.66% (9/542), 15.3% (83/542), 6.08% (33/542), 13.2% (72/542), and 9.22% (50/542) among ORF5-positive farms, respectively. The recent isolates of lineage 1 were grouped with highly virulent viruses of the United States (NADC30 virus, MN184, etc.) and China (Fig. 1c). The recent isolates of lineage 5 were grouped with 57 Korean viruses, three Korean reference strains (CA-1, PL97-1, and LMY) isolated in 2005–2009 [36], and VR2332, Ingelvac PRRS MLV, and so on. Meanwhile, three Korean lineages (kor A, B, and C) formed their own group separate from PRRSVs isolated from foreign countries. Importantly, lineage kor B was grouped only with recent field viruses isolated in 2014–2016, while lineages kor A and C included 24 and 32 field viruses isolated in 2003–2010, respectively [32, 36]. NT (AA) sequence similarity rates to VR2332 were 82.3–86.9% (81.7–87.6%), 87.6–99.3% (85.6–99.0%), 85.1–89.1% (82.1–87.6%), 85.7–89.1% (83.6–90.1%), and 85.2–86.4% (83.6–86.1%) for lineages 1 and 5 and lineages kor A, B, and C of field viruses isolated in 2013–2016, respectively. Meanwhile, NT (AA) sequence similarity rates to VR2332 were 88.9% (88.1%), 88.9–99.5% (88.1–99.0%), 87.6–89.6% (82.6–88.6%), and 84.7–87.1% (84.1–88.6%) for lineages 4 and 5 and kor A and C of field viruses isolated in 2003–2010, respectively. Regarding sequence similarity to vaccine strains, NT (AA) sequence similarity to Ingelvac PRRS MLV was highly similar to VR2332 for field viruses isolated in 2013–2016 as well as in 2003–2010. NT (AA) sequence similarity to P129 was 85.2–86.6% (84.6–86.6%), 86.7–91.7% (84.6–91.5%), 85.6–89.2% (85.1–90.1%), 86.1–89.1% (85.1–89.1%), and 82.6–86.9% (81.6–87.6%) in lineages 1 and 5 and kor A, B, and C of field viruses isolated in 2013–2016, while 89.7% (89.1%), 88.2–91.9% (88.1–92.5%), 87.2–90.2% (86.1–93.0%), and 85.4–88.1% (84.1–89.1%) in lineages 4 and 5 and kor A and C of field viruses isolated in 2003–2010, respectively (Table 2B).

### NEs and N-glycosylation sites of ORF5 amino acid sequences

**Fig. 3:**
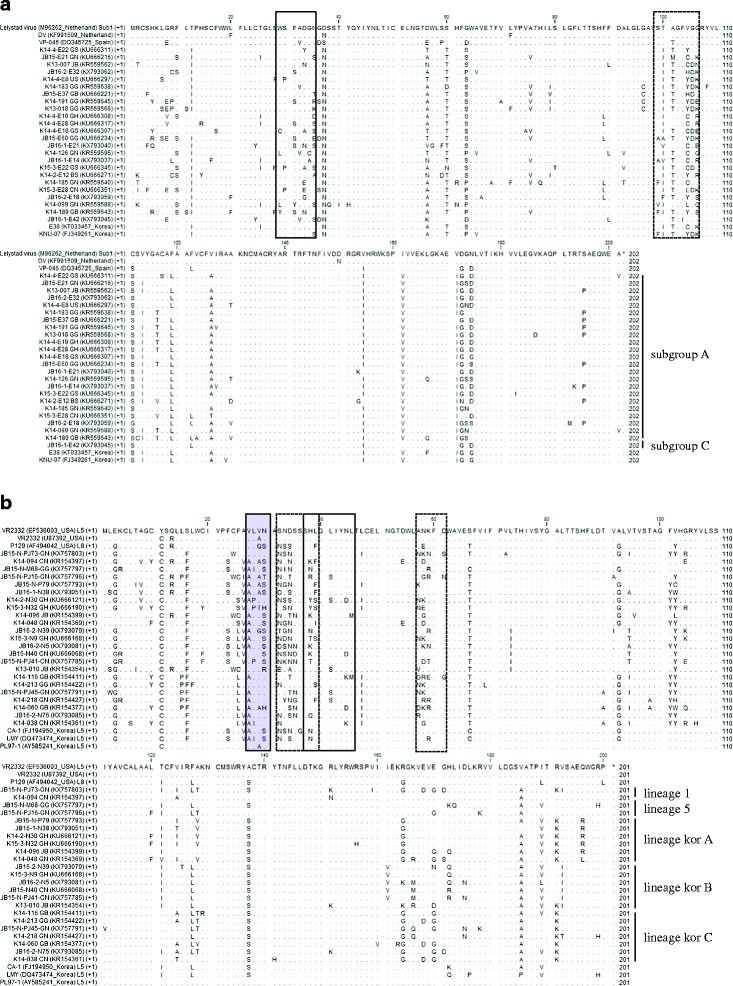
Multiple alignment of ORF5 amino acid sequences of representative isolates in each subgroup (Type 1 PRRSV) (**a**) and lineage (Type 2 PRRSV) (**b**). The representative isolates were selected based on main amino acid sequence of neutralizing epitope of Type 1 PRRSV (residues 29–35) [23] and Type 2 PRRSV (residues 37–45) [37]. The solid line box and gray color box represent neutralizing epitope and decoy epiotpe (residues 27–31) [57], respectively. The dot-line box indicates hypervariable regions [58]

NT sequence deletions or additions in the ORF5 sequence were not observed in the Korean isolates. Korean Type 1 PRRSV showed 7 and 25 different AA sequences of NEs (residues 29–35) [23] for field viruses isolated in 2005–2009 and 2013–2016, respectively. For Type 2 PRRSV, 12 and 23 different AA sequences in NEs (residues 37–45) [37] were observed for Korean field viruses isolated in 2003–2010 and 2013–2016, respectively. NEs (WSFADGN for DV or Lelystad, WSFVDGN for VP-046, SHLQLIYNL for Ingelvac PRRS MLV, or VR2332 and SHFQLIYNL for P129) of vaccine or PRRSV prototype strains (VR2332 and Lelystad) were compared with those of field isolates (Table 3). NEs of vaccine or PRRSV prototype strains were observed in 91.4% (74/81) and 80.0% (236/295) of Type 1 PRRSV field isolates of 2005–2009 and 2013–2016, and 67.5% (77/114) and 61.1% (151/247) of Type 2 PRRSV field isolates of 2003–2010 and 2013–2016, respectively. The NE (WSFADGN) of DV or Lelystad appeared as a main epitope in subgroups A, B, and C for all Korean Type 1 PRRS isolates, while the NE (SHLQLIYNL) of Ingelvac PRRS MLV or VR2332 was observed as the primary epitope of all Type 2 PRRSV lineages except for kor A and B of field isolates of 2013–2016, in which the NE (SHFQLIYNL) of P129 was the primary epitope. Regarding the pattern of N-glycosylation (number and sites of N-glycosylation), putative N-glycosylation sites were found at residues 37, 46, and 53 among the majority of Type 1 PRRS field isolates, resulting in 37-46-53 as the main N-glycosylation pattern throughout all subgroups in 2005–2016. In contrast, even if the putative N-glycosylation sites were highly conserved at residues 44 and 51 for Type 2 PRRS field isolates, the N-glycosylation pattern was significantly variable regardless of isolation year and lineage: 4–5 sites (residues 30, 33, 34, 44, 51, and 59) for lineage 1, 3–5 sites (residues 30, 32–35, 43, 44, 50, and 51) for lineage 5, 2–4 sites (residues 32–35, 44, and 51) for lineage kor A, 2–4 sites (residues 32–35, 43, 44, 50, and 51) for lineage kor B, and 2–5 sites (residues 30, 32–35, 44, and 51) for lineage kor C. Although the N-glycosylation pattern of Ingelvac PRRS MLV (30-33-44-51) was identified as the main pattern in lineages 1 and 5 of field viruses isolated in 2003–2010, novel N-glycosylation patterns appeared highly dominant overall. The N-glycosylation pattern (34-44-51) of VR2332 was identified in all lineages of Korean field viruses isolated in 2003–2016, while the N-glycosylation pattern (46-53) of Lelystad was not identified in all field isolates. Many of the AA changes were observed in hypervariable regions of Korean Type 1 (residues 99–106) and Type 2 (residues 32–39 and 57–61) field viruses isolated in 2013–2016 (Fig. 3).

**Table 3:** Diverse neutralizing epitopes and putative N-glycosylation sties on GP5 of Korean field PRRSVs isolated in 2003–2016 and commercial vaccine strains

Type	Vaccine strain/Isolated year	Subgroup /Lineage	AA sequence of neutralizing epitopes	AA position for putative N-glycosylation sites
1	DV	subgroup C	WSFADGN	37-46-53
	VP-046	subgroup A	WSFVDGN	35-46-53
	2005-2009	subgroup A	WSFADGN(71)^a^, WSFVDGN(1), WSFADGS(3), WSFASGN(1), WSFADCN(1)	37-46-53(75), 37-53(2)
		subgroup B	WSFADGN(2), WSFANGN(1), WSFVDGS(1)	37-46-53(3), 37-53(1)
	2013-2016	subgroup A	WSFADGN(214), WSFAYGN(1), WSFVDGN(5), WSFADGT(7), WSFADGS(11), WSSADGR(1), WSFADGK(2), WSFANGN(1), WSFADGD(2), WSSADGS(3), WPFADGN(4), WSSADGN(5), CSFAAGS(4), WSSADGE(1), WSFADGA(1), WSFADAN(1), WPFAAGS(3), WSFAGGN(2), WSFAEGN(1), WSSANGS(1), LSFVDCN(1), WPFAEGN(1), YSSANGN(1), LSYADGS(1)	37-46-53 (252), 37-53 (12), 35-46-53 (2), 33-37-46-53 (1), 37-46-53-61(2), 37-38-46-53(2), 46-53(1), 36-46-53(1), 46 (1)
		subgroup B	WSFANGN(1)	37-46-53(1)
		subgroup C	WSFADGN(15), WSFVDGN(1), WSFVDGS(2), WSFADGS(1), WSFANGN(1)	37-46-53(14), 35-46-53(4), 37-53(2)
2	IngelvacPRRS MLV	lineage 5	SHLQLIYNL	30-33-44-51
	P129	lineage 8	SHFQLIYNL	32-44-51
	2003-2010	lineage 4	SHLQLIYNL	30-33-44-51
		lineage 5	SHLQLIYNL(43), SNLQLIYNL(11), SKFQLIYNL(1), XNLQLIYNL(1), SNLQSIYNL(1)	30-33-44-51(17), 34-44-51(17), 32-33-44-51(7), 33-34-44-51(3), 32-44-51(2), 30-35-44-51(1), 30-33-43-50(1), 30-33-34-44-51(4), 30-34-44-51(3), 30-34-35-44-51(1), 33-44-51(1)
		lineage kor A	SHFQLIYNL(9), SHLQLIYNL(1), SKFQLIYNL(1), SKIQLIYNL(2), SHIQLIYNL(1), SKLQSIYKL(1), SNLQLIYNL(2), SNFQLIYNL(4), SKLQLIYNL(3)	32-44-51(5), 33-44-51(5), 33-51(1), 32-33-44-51(3), 32-43-50(1), 35-43-50(1), 33-43-50(3), 30-33-44-51(1), 34-44-51(2), 33-34-44-51(1), 30-35-44-51(1)
		lineage kor C	SHLQLIYNL(24), SHLQSIYNL(8)	30-34-44-51(13), 34-44-51(3), 30-44-51(1), 30-33-44-51(2), 33-34-44-51(5), 30-33-34-44-51(4), 30-35-44-51(2), 33-44-51(2)
	2013-2016	lineage 1	SHLQLIYNL(9)	30-33-34-44-51(4), 30-34-44-51(1), 34-44-51-59(2), 33-34-44-51(1), 30-33-44-51(1)
		lineage 5	SHLQLIYNL(77), SKFQLIYNL(2), SNLQLIYNL(3), SRLQSIYNL(1)	30-33-44-51(48), 32-35-44-51(1), 33-34-44-51(4), 30-33-34-44-51(10), 34-44-51(7), 30-33-43-50(2), 30-44-51(2), 30-35-44-51(4), 32-33-44-51(3), 33-44-51(2)
		lineage kor A	SHFQLIYNL(14), SHLQLIYNL(2), SKFQLIYNL(2), SNLQLIYNL(1), SYSQLIYNL(4), SYSQSIYDL(1), SYSQSIYNL(1), SKLQLIYNL(4), SKLQLIYNM(1), SNFQLIYNL(2), SHIQLIYNL(1)	34-44-51(17), 33-44-51(3), 32-33-44-51(6), 32-44-51(3), 32-35-44-51(2), 33-34-44-51(1), 32-51 (1)
		lineage kor B	SNLQLIYNL(47), SHLQLIYNL(2), STLQLIYNL(12), STSQLIYNL(1), SKLQLIYNL(7), SNPQLIYNL(1), SKLQLIYDL(1),SHLQSIYNL(1)	34-44-51(39), 34-43-50(2), 34-35-44-51(6), 33-44-51(4), 34-51(1), 33-44-51(1), 32-44-51(8), 33-43-50(1), 33-34-44-51(9), 32-33-44-51(1)
		lineage kor C	SHLQLIYNL(38), SHLQLIYKM(1), SHLQSIYNL(4), SHSQLIYNL(1), SHFQSIYNL(1), SHLQLIYDL(1), SQLQLIYNL(3), SHLQLIYKL(1)	30-33-44-51(8), 30-35-44-51(6), 34-44-51(5), 30-34-44-51 (4), 32-35-44-51(1), 32-33-44-51(2), 33-51(1), 30-34-35-44-51 (4), 33-34-44-51(2), 35-51(1), 34-35-44-51(3), 33-44-51 (6), 30-33-51(1), 30-33-34-44-51(5), 35-44-51(1)

^a^Main neutralizing epitope and pattern of N-glycosylation site at each subgroup or lineage were underlined

## Discussion

In this study, the recent geographic prevalence and genetic characteristics of Korean PRRSVs were investigated using field isolates circulating in swine farms nationwide. RT-PCR of 631 clinical samples (farms) revealed that 38.4% (242/631) were infected by Type 1 PRRSV, 37.4% (236/631) were infected by Type 2 PRRSV, and 24.2% (153/631) were simultaneously infected by both Type 1 and Type 2 PRRSV. When compared with a previous report [38], in which the single infection rates of Type 1 or Type 2 PRRSV and co-infection of both types in swine farms were 29.4, 54.4, and 16.2%, respectively, it was noted that Type 1 PRRSV has become highly prevalent in the 10 years following its first isolation in 2005. In addition, the increased number of farms infected with both Type 1 and Type 2 PRRSV suggested that effective control against PRRS in the Republic of Korea might have to be implemented with the use of control measures against both genotypes.

All of the Korean Type 1 PRRS viruses were classified into the subgroups A, B, and C of subtype 1, consistent with a previous report [34]. The majority of the Korean Type 1 PRRS isolates (274 isolates from 2013 to 2016 and 77 isolates from 2005 to 2009) belonged to subgroup A, showing close relatedness with a Spanish strain, CRESA11. Subgroup B viruses (one isolate from 2013 to 2016 and four isolates from 2005 to 2009) were grouped with a Thai strain (03RB1) and pan-European strains. Subgroup C viruses (20 isolates from 2013 to 2016) were grouped with Type 1 PRRS live vaccine strains (DV and VP-046) and other pan-European strains. Lee et al. [39] first reported that a Korean isolate was grouped with DV. However, it is not possible to define genetic relatedness between the field isolate and the vaccine strain due to the lack of sequence information. Subgroup C viruses (5 DV- and 15 VP-046-like strains) isolated in this study showed a phylogenetic closeness to the vaccine strains with greater than 99% NT sequence similarity without geographical predominance. Therefore, it has been suggested that the vaccine-like viruses originated from the Type 1 PRRS live vaccines commercialized in 2014 and would become highly prevalent over time in the Republic of Korea.

Korean Type 2 PRRS viruses have been divided into lineages 1, 4, and 5, as well as the Korean lineages kor A, B, and C based on global PRRSV phylogeny [30]. The majority of the Korean Type 2 PRRS field isolates belonged to lineage 5 and the Korean lineages. Lineage 5 viruses were grouped with VR2332-like viruses genetically close to the Ingelvac PRRS MLV vaccine virus, which has been used commercially since 1996. The lineage also included Korean reference field strains, PL97-1 [40], LMY [32, 33], and CA-1 [41]. Regarding the emergence of novel Korean lineages, lineages kor B and C were first classified in this study, while lineage kor A was first reported as a Korean lineage in a previous study of field isolates of 2010 [36]. Lineages kor A and C were observed to include field viruses isolated in 2005–2009 and 2013–2016, which indicated that these groups have developed genetic components geographically distinct from those in foreign countries for longer than 10 years. Meanwhile, lineage kor B comprised only recent isolates that originated from Gyeongnam Province between 2014 and 2016, suggesting that this group may have recently established regionally unique genetic properties within the Republic of Korea.

Nine viruses of lineage 1 isolated between 2014 and 2015 were closely related to NADC30, with 92.3–94.9% NT sequence similarity to NADC30, which was one of the representative virulent strains along with MN184 and SDSU73 in the United States [42]. Chinese NADC30-like viruses (HNjz15, JL580, HENAN-HEB, HENAN-XINX, and CHsx140) were also reported to cause obvious clinical symptoms in pigs with virulence comparable to that of NADC30 [43–45]. It should be noted that the circulation of NADC30-like viruses in the Republic of Korea has not been previously reported. Although the virulence of NADC30-like viruses is known to be less than that of highly pathogenic PRRS viruses [43], there is a strong need to define the virulence of recent NADC30-like isolates in pigs in Korean farms. There are two possible explanations for the recent emergence of NADC30-like viruses. First, the NADC30-like viruses may have recently been introduced by the importation of breeding pigs, likely as Chinese NADC30-like viruses [45]. Second, it has been assumed that NADC30 was introduced 7–10 years ago, circulated in a particular environment (e.g., with wild boars as hosts) based on an estimated mutational change rate (0.78% per year) of ORF5 NTs identified in previous studies [24, 46], and was recently transmitted to domestic swine farms in various ways (e.g., wild boar farming).

The NEs and N-glycosylation patterns of GP5 were highly variable, and novel epitopes and N-glycosylation patterns continuously emerged and disappeared in the field viruses, although this was more apparent in field viruses isolated in 2013–2016 than in 2003–2010 (Table 3). As expected, NEs of vaccine strains or PRRSV prototype strains existed in the majority of field isolates (82.2 and 61.0% for Type 1 and Type 2 PRRSV, respectively) without a preference for a certain subgroup or lineage. In particular, NEs of DV (or Lelystad) (WSFADGN) and Ingelvac PRRS MLV (or VR2332) (SHLQLIYNL) were maintained in approximately 80.4 and 54.6% of field isolates, respectively. It has been speculated that the dominance of the two NEs might be due to extensive vaccination using vaccine strains (Ingelvac PRRS MLV and DV) that originated from PRRSV prototype strains. Nevertheless, it has been noted that the emergence of novel NEs has been significantly expedited over time in PRRSV field isolates (8.6% of 2005–2009 field isolates vs. 20.3% of 2013–2016 field isolates for Type 1 PRRSV; 31.8% of 2005–2009 field isolates vs. 42.3% of 2013–2016 field isolates for Type 2 PRRSV). Regarding the N-glycosylation pattern, it was interesting to note that N-glycosylation sites were highly diverse and changeable in Korean Type 2 PRRS field isolates, while being conserved in Korean Type 1 PRRS field isolates. NEs and the N-glycosylation pattern are closely associated with virus propagation, neutralization, and immunological protection [47–51]. Therefore, it has been suggested that the strong preference for mutation of NEs and the N-glycosylation pattern, particularly in Type 2 PRRSV, might be associated with limited protective efficacy of PRRS vaccination against heterologous viruses in the field, as shown in previous studies [52–54].

Type 2 vaccine-like viruses have become highly prevalent since the first use of Ingelvac PRRS MLV in 1996 [32]. In this study, lineage 5, including the Type 2 vaccine strain and vaccine-like viruses, showed the highest prevalence (15.3%) in the field. Likewise, the recent prevalence of subgroup C, including Type 1 vaccine-like viruses, is low (3.69%). However, extensive use of Type 1 live vaccines may result in a dramatically increased prevalence of Type 1 vaccine-like viruses and/or isolation of field viruses sharing a genomic part of Type 1 live vaccine strains in the near future, caused by genetic evolution mechanisms of PRRSV, random point mutations, and homologous recombination [55]. To minimize the circulation of live vaccine strains in the field, we may have to strengthen swine farm bio-security to prevent contamination of live vaccine strains among swine farms vaccinated with live vaccines. At the same time, limited use of live vaccines may be recommended in young pigs (3–8 weeks old) or pregnant gilts/sows of PRRS-affected farms.

## Conclusions

This study suggests that PRRS viruses in the Republic of Korea have recently developed genetic characteristics geographically distinct from currently prevalent global PRRS viruses or commercial vaccine strains. Therefore, further study will be required to evaluate how the established genetic characteristics have affected PRRS outbreaks in domestic swine farms, which will provide important information for the implementation of control and preventive measures in swine farms.

## Abbreviations

AA: Amino Acid
NE: Neutralizing epitope
NT: Nucleotide
ORF: Open Reading Frame
PRRSV: Porcine reproductive and respiratory syndrome virus
